# Polyphasic Characterization of *Brucella* spp. in Livestock Slaughtered from Abattoirs in Eastern Cape, South Africa

**DOI:** 10.3390/microorganisms12010223

**Published:** 2024-01-22

**Authors:** Koketso Desiree Mazwi, Francis Babaman Kolo, Ishmael Festus Jaja, Charles Byaruhanga, Ayesha Hassim, Henriette van Heerden

**Affiliations:** 1 Department of Veterinary Tropical Diseases, Faculty of Veterinary Science, University of Pretoria, Onderstepoort, Pretoria 0002, South Africa; 2 Department of Livestock and Pasture Science, Faculty of Science and Agriculture, University of Fort Hare, Alice 5700, South Africa; 3 Department of Agriculture and Animal Health, University of South Africa, Roodepoort, Johannesburg 1709, South Africa; 4 National Agricultural Research Organisation, Entebbe P.O. Box 259, Uganda

**Keywords:** Brucellosis, livestock, tissue samples, culture positive, AMOS-PCR, *Brucella abortus*, *B. melitensis*, South Africa

## Abstract

In livestock, brucellosis is mainly an asymptomatic disease except when abortion occurs; therefore, two serological tests are used for diagnosis as no single test is suitable. Abattoir samples enable a combination of culture, molecular, and serological tests to detect brucellosis. This study assessed *Brucella*-specific PCR (ITS-PCR) to detect brucellosis and to conduct a molecular characterization of *Brucella* spp. isolated from PCR-positive livestock (n = 565) slaughtered at abattoirs and the appropriate sample tissue(s). ITS-PCR detected *Brucella* DNA in 33.6% of cattle, 14.5% of sheep, and 4.7% of pig tissues. Impure *Brucella* cultures from PCR-positive tissues were 43.6% (44/94) of cattle, 51.7% (15/29) of sheep, and 50% (2/4) of pigs with predominantly *B. abortus* identification with AMOS-PCR and low isolation of mixed *B. abortus* and *B*. *melitensis* in all species. In cattle, 33% of isolates were from lymph nodes, while in sheep 38.0% were from the liver and kidney and only from tonsils in pigs (2/4). *Brucella* infections identified with AMOS-PCR were present in seropositive and mainly seronegative (75.6–100%) livestock with the potential to cause brucellosis during pregnancy or breeding. This study demonstrated the value of the polyphasic approach, especially with chronic infections and the potential risk of these asymptomatic animals.

## 1. Introduction

Brucellosis is a highly contagious zoonotic infection of humans, domestic, and marine animals [1]. The *Brucella* species are facultative intracellular Gram-negative, non-spore forming, cocco-bacilli bacteria [2,3,4] causing a disease called brucellosis. In animals, *Brucella* invades the host without any clinical symptoms, resulting and progressing to acute infection only when the bacteria replicate actively within the macrophages and other replication sites [4]. The infection in animals remains asymptomatic in most cases, or until the first pathological signs/symptoms appear [4]. Chronic infections occur when the bacterial load decreases after plateauing, with sporadic clinical symptoms when the infection localizes in the reproductive system of sexually mature animals, resulting in sterility in males and placentitis as well as abortion in females during pregnancies. It continues to spread amongst animals in the herd [5,6,7,8]. Infection is described as mostly self-limiting [8] due to low activation of phagocytosis and other host defences related to innate immunity [9,10]. After the initial phase of the illness has passed, the majority of brucellosis symptoms are not pathognomonic, and the organism can persist over time in the mammary glands and supramammary lymphatic nodes of 80% of infected animals [11]. *Brucella* replicates extensively in the endoplasmic reticulum (ER) compartment within the host cells [12]. The host cells’ specialized compartment where intracellular pathogens reside prevents antibiotics from reaching them, thus affecting the efficacy of current therapies [12]. The association of *Brucella* within the host cell ER provides optimal bacterial growth conditions and replications in organs such as the spleen, lymph nodes, liver, bone marrow, epididymis, and placenta, which is rich in reticuloendothelial cells [8]. In the chicken embryo model, the replication of *Brucella abortus* spreads to all tissues, with the liver and spleen being the most severely infected [13].

Gonzalez-Espinozo et al. [8] reviewed investigations to improve cultures other than blood, such as bone marrow aspirates, liver, and lymph nodes, based on the rationale to obtain specimens from macrophage-rich tissues where *Brucella* organisms multiply and concentrate as this may increase bacterial recovery. Culture from these tissues remains far from resolved due to its non-specific signs and symptoms that are comparable to other febrile diseases, its slow growth rate on culture, and the complexity of its sero-detection, and thus brucellosis remains difficult to diagnose [14,15]. The sensitivity of culture depends on the disease stage, *Brucella* spp. culture medium and the technique being used, the quality of circulating bacteria, and the number of contaminants present in the sample [16]. The skin, hair, limbs, blood, stomach, gut contents, bile, and other excretions of the animal, as well as the facilities, can all contaminate the sample taken from the carcasses of the animals throughout the slaughter process in the abattoirs [17]. It is crucial to minimize any surface contamination occurring in the abattoirs during the slaughtering process, using a hazard analysis critical control point (HACCP) plan, in order to effectively handle and regulate the microbiological hazards connected with meat products [18].

Several molecular and high-resolution phenotypic assays that allow the differentiation of *Brucella* spp., the biovars, and the traceability of the source have been published [19,20]. However, only the gold standard (culture) is capable of reliably diagnosing brucellosis [21,22]. *Brucella abortus* and *B. melitensis* isolates take up to 4–7 days for growth in the laboratory; however, an increased number of contaminants including fungi and bacteria are usually observed, resulting in the regular contamination of culture plates and the decreased sensitivity of bacteriological diagnosis [23]. The most common sample for brucellosis diagnosis is serum for serological tests, which is not an appropriate sample for culture. Investigation at abattoirs where various sample types can be collected offers a polyphasic approach. Rose Bengal test (RBT) is used as a serological screening test with high sensitivity and low specificity resulting in false positives. RBT positive samplescan be confirmed through a compliment fixation est (CFT) and/or indirect enzyme-linked immunosorbent assay (iELISA), which both have high specificity, but the CFT is less sensitive than iELISA, resulting in false-negative results [24]. Modern molecular approaches are currently not widely implemented in low-income nations where brucellosis is endemic in livestock [14,25]. This may be due to resource constraints. However, *Brucella* genus-specific PCR assays (conventional and real-time) such as 16–23S ribosomal DNA interspacer (ITS) region, bcsp31 and IS711-based assays have been used as well as multiplex PCR assays, namely AMOS- PCR for *B. abortus* bv 1, 2 and 4, *B. melitensis* bv 1–3, *B. ovis*, and *B. suis* bv 1 and Bruce-ladder PCR, which identify all *Brucella* spp. [26].

The *Brucella* genus currently consists of twelve species of which four species are pathogenic to humans [27]. *Brucella melitensis* and *B. abortus* commonly infect small ruminants and cattle, respectively, leading to abortions and infertility thus resulting in significant economic losses [28]. Five *Brucella* spp. have been discovered in wildlife and marine mammals, while four additional distinct strains have been discovered in rodents, frogs, baboons, and humans [29]. The most pathogenic species for human brucellosis is *B. melitensis*, followed by *B. suis*, and then *B. abortus* [29]. *Brucella ceti*, *B. inopinata*, and *B. canis* (rarely) are also known to cause human brucellosis [29].

Brucellosis-infected animals are the primary cause of human brucellosis, a persistent illness with serious side effects if neglected [30]. Despite brucellosis being a notifiable disease in many countries, official statistics do not accurately reflect the number of cases that are reported each year [31]. Most developing countries in Africa have listed brucellosis as an under-reported endemic infection; due to the limited number of studies and the lack of epidemiological data [25]. In South Africa (SA), *B. abortus* and *B. melitensis* have been reported in humans, cattle, sheep, and goats at the turn of the century [32,33]. The cattle population contributes to the majority of the income in SA and thus bovine brucellosis has a significant negative economic impact on the country’s dairy and beef industries [34]. A nationwide bovine brucellosis scheme existed in SA since 1979 [35] and includes the vaccination of heifers and test and slaughter of high-risk bovines such as dairy and export. This scheme is voluntary for other animal owners and depends on the resources and willingness of the owners [36]. Most owners are aware that a positive test results in quarantine, which limits participation in this scheme focusing mainly on high-risk bovines as well as knowledge about brucellosis seroprevalence amongst livestock in SA. Despite the scheme, bovine brucellosis seroprevalence has increased from 3.74% to 9.18% based on retrospective results reported in SA from 2007 to 2015 [37]. The aim of this study was to use a polyphasic approach to (I) screen tissue samples using *Brucella* ITS-PCR to detect *Brucella* DNA; (II) isolate *Brucella* from ITS-PCR-positive tissues using a selective medium; (III) assess the most appropriate sample type (lymph nodes, spleen, kidney, liver, and tonsils (the latter only from cattle and pigs)) to isolate *Brucella*; and (IV) characterize *Brucella* spp. isolates using AMOS-PCR and Bruce-ladder multiplex PCR assays from seropositive and seronegative livestock (cattle, sheep, and pigs) slaughtered at abattoirs in the Eastern Cape Province, SA.

## 2. Materials and Methods

### 2.1. Description of the Study Area

This study was based on voluntary participation from abattoirs in the Eastern Cape Province, SA. The *Brucella* isolates characterized in this study were recovered from cattle, pigs, and sheep (lymph nodes, liver, spleen, kidney, and tonsils (the latter from cattle and pigs)) collected from the abattoirs. The Eastern Cape (at 168,966 km^2^) has the largest percentage of livestock in the country [38] and stretches along the Indian Ocean between Western Cape and KwaZulu-Natal provinces. The collection of samples was from five abattoirs in the Eastern Cape Province, but the livestock slaughtered were not only from the Eastern Cape but included livestock transported from bordering provinces such as KwaZulu-Natal and Free State. The latter two provinces do not have any movement control for livestock. In SA, Western Cape is the only province that enforces movement control of foot and mouth disease-susceptible animals into and out of the province [39].

### 2.2. Study Design and Sample Size

The abattoirs recruited for this study included both high-throughput and low-throughput abattoirs. The target animal population was sheep, cattle, and pigs and included apparently healthy animals with unknown *Brucella* status. During the abattoir visits, blood (serum) and tissue (kidney, spleen, liver, tonsils, and lymph nodes) samples were collected from corresponding animals. For this study, tissue samples were collected from 565 animals, comprising 280 cattle, 200 sheep, and 85 pigs. This number may not represent the population ratio of 12.7 million cattle, 22.3 million sheep, and 1.4 million pigs in SA (https://www.agriseta.co.za/wpcontent/uploads/2021/02/Agriseta_Red_Meat_SSSP_DIGITAL.pdf, accessed on 13 November 2023) because the number of animals sampled was dependent on the number slaughtered at the abattoir on the day of visit. The sample size was calculated using the following formula n = z^2^P_exp_Q/L2, where n is the sample size, P_exp_ is the expected prevalence, and L is the precision of the estimate (also called “the allowable error” or margin of error), which is taken to be 0.05 for this study. Q = 1 − P_exp_, and Z is the (1 − α2) percentile of a standard normal distribution [40]; for α = 0.05, Z = 1.96. In order to estimate the sample size, 11% prevalence was applied based on recent data from other South African provinces [41] due to a lack of recent data on *Brucella* seropositivity in Eastern Cape province. This resulted in a sample size of 151 for each species, which adheres to the sample size per the calculation, except the porcine samples, since the abattoirs received fewer pigs during the sampling period. Animals were sequentially sampled using a randomly selected subset of a single species. Samples were collected in a sterile plastic bag and stored at −20 °C at the University of Pretoria, Department of Veterinary Tropical Diseases Biosafety Level 2+ laboratory prior to processing.

### 2.3. Sample Collection Procedure

**Figure 1:**
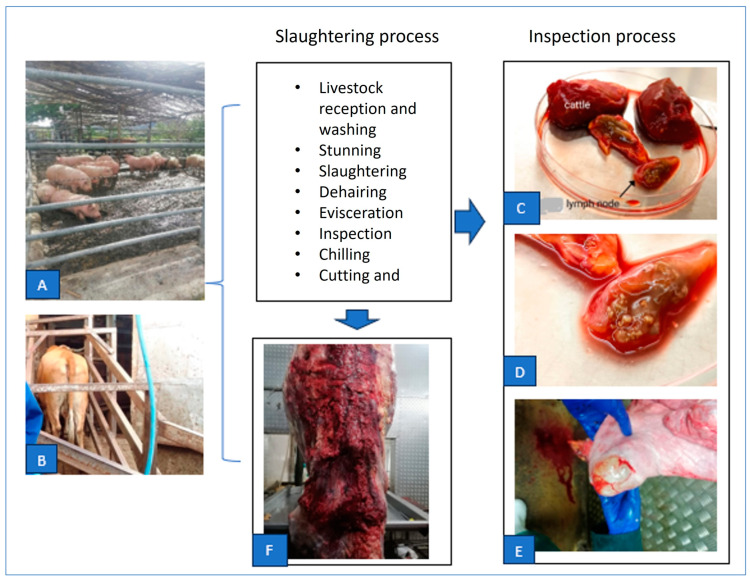
Collection of samples during slaughtering process workflow and inspection in this study. (**A**,**B**): Livestock in holding pens at the abattoirs. (**C**) Processing of approximately 100 g of each tissue (**D**) Atypical cattle lymph node with visible lesions. (**E**) Cyst/abscess on a liver. (**F**) Bruised carcass post slaughter.

An opportunistic sampling procedure was followed for the collection of the samples. Multiple animal species are slaughtered in these selected abattoirs on any given day. Animals were sampled consecutively from within a randomly selected subset of a single species. That is, for every species, the daily quota of animals was sampled one after the other to ensure the accurate sampling and assignation of samples per species. Our approach was carried out in three steps: (I). Planning: The relevant information was requested from the abattoir managers regarding the animals and herd information, and this included the age, sex, vaccination status, location, and owners or seller of the animals. However, the animals and herd vaccination information were not available, and only the abattoir and regional veterinary services are allowed access to the location and owner information, which must be requested through official procedures by the regional state veterinary office which were obtained. (II). Sample collection: The selected abattoirs in the Eastern Cape Province are located more than 100 km apart, except for two which are located within the same region. We aimed to collect samples from herds as *Brucella* infection is a known herd disease [42]. Upon the slaughtering of animals by butchers, the animals were immediately eviscerated, and all the organs were removed from the carcasses (Figure 1A,B). To avoid animal-to-animal contamination, the knifes were cleaned with boiling hot water between uses. Approximately 100 g of each tissue was excised (Figure 1C). The sample collection procedure was lengthy, since it also included a cursory meat inspection by our team and the abattoir meat inspector (Figure 1D–F). Therefore, only one abattoir could be sampled per day. The tissues were stored in a clearly labelled sterile plastic bag followed by ~4 °C cold chain in the abattoir. (III). Packing and transportation: The samples were stored in a −20 freezer before transportation to the University of Pretoria, Department of Veterinary Tropical Diseases, SA, in triple-layer packaging for processing in accordance with the National Road Traffic Act, 1996 (Act No. 93 of 1996).

### 2.4. Sample Processing

The excised tissues were processed according to set laboratory protocols in a bio-safety level (BSL) 2 plus laboratory. The kidney, spleen, liver, tonsils, and lymph nodes were examined for lesions and calcification. A cubic centimetre of healthy-looking tissue was dissected with a sterile surgical blade and aliquoted into two separate cryovials. These matching tubes were submitted for direct DNA extraction, PCR, and microbial isolation, respectively. The serological test results were determined by [43] using serum samples; these were subjected to the RBT from Onderstepoort Biological Products, Pretoria, South Africa, CFT (conducted at Onderstepoort Veterinary Institute laboratory where the test is SANAS accredited for bovine, but not sheep and pigs), and the iELISA (IDVet, Grabels, France) as per the manufacturer’s recommendations.

### 2.5. Genomic DNA Extraction

DNA was extracted directly from all the tissue samples for *Brucella* spp. screening. This was performed using the Pure-Link Genomic DNA Kit (tissue protocol) according to the instructions of the manufacturer (Thermo Fisher Scientific, Waltham, MA, USA).

### 2.6. Brucella Genus PCR Screening Using ITS

DNA amplification for the detection of the target *Brucella* gene using genus-specific 16S-23S rRNA interspacer region (ITS) primers (ITS66: ACATAGATCGCAGGCCAGTCA and ITS279: AGATACCGACGCAAACGCTAC) was used for the detection of *Brucella* DNA in the tissues [44]. During the culturing process, suspected *Brucella* isolates were screened with ITS-PCR to detect *Brucella* spp. DNA. Briefly, a PCR master mix of 12 μL was prepared as follows: 6.5 μL Dream Taq polymerase, 0.3 μL (0.2 μM) Forward primer, 0.3 μL reverse primer (0.2 μM) and 4.9 μL of nuclease-free water (Thermo Fisher Scientific, Johannesburg, South Africa). From each sample, 3 μL of DNA was used in a 15 μL PCR reaction. The mix was amplified on a thermal cycler (Veriti, Thermo Fisher Scientific, Waltham, MA, USA) with a heated lid, preheated to 105 °C. The PCR cycling condition consisted of 95 °C for 3 min, followed by 35 cycles of 95 °C for 1 min, 60 °C for 2 min, 72 °C for 2 min, and a final extension of 72 °C for 5 min. The target DNA had a product size of 214 bp which was observed on agarose electrophoresis. The positive controls used were *B. abortus* bv 1 strain (BCCN R4) and *B. melitensis* Rev 1 (Onderstepoort Biological Products, Pretoria, South Africa). The amplified products were examined by electrophoresis in a 2% agarose gel (agarose LE) (Lasec SA (Pty), Midrand, South Africa) and stained with ethidium bromide (0.03 μL/mL). The gel ran at 120 volts for 1 h. The gel was documented under UV light by a molecular imager (Bio-rad, ChemiDoc^TM^ XRS, Hercules, CA, USA).

### 2.7. Sample Preparations, Brucella Culture, and Bacteriological Examination

Each tissue was homogenized with 1 mL of ddH_2_O in a Precellys 24 lysis and tissue homogenizer (Bertin technologies, Paris, France). About 200 μL of the tissue homogenate from pre-screened *Brucella* ITS-PCR-positive tissues was inoculated onto the modified CITA medium [45] and incubated at 37 °C with 5.0% CO_2_ for 5–14 days, followed by subculturing for purification where necessary. Culture plates were considered negative and discarded following 14 days of incubation with no growth observed. *Brucella* suspected isolates were selected based on their morphology on the CITA medium, Gram staining, and modified Ziehl–Neelsen staining [40]. DNA was extracted from all the *Brucella* suspected (Gram-negative and modified Ziehl-Neelsen) isolates for molecular characterization using the Pure-Link Genomic DNA Kit (Gram-negative protocol). During purification, suspect *Brucella* single colonies were transferred to CITA medium and screened using staining and/or ITS-PCR. Fast-growing bacteria kept on overgrowing slow-growing *Brucella* colonies observed with Gram staining and ITS-PCR resulted in the impure *Brucella* isolates. Gram-negative fast-growing isolates were selected on the culture plates and submitted for genomic DNA extraction (Pure-Link Genomic DNA Kit; Section 2.5) and 16S sequencing (see Section 2.8). These fast-growing contaminants grew on modified CITA medium in the presence of antibiotics (natamycin, nitrofurantoin, amphotericin B, colistin, nystatin, and vancomycin). AMOS-PCR assay was used to identify *Brucella* spp. from DNA extracted (Pure-Link Genomic DNA Kit; Section 2.5) from impure *Brucella* culture isolates from livestock tissues (see Section 2.10).

### 2.8. Identification of Fast-Growing Contaminants on Culture

Metagenomic analyses of full-length 16S gene amplicons were conducted by Inqaba biotec, Pretoria, SA. Isolated DNA samples were sequenced on the Sequel system using PacBio (www.pacb.com). Raw sub-reads were processed through the SMRTlink (v11.0) Circular Consensus Sequences (CCS) algorithm to produce highly accurate reads (>QV40). These highly accurate reads were processed through DADA2 (https://benjjneb.github.io/dada2/index.html) and qiime2 (https://docs.qiime2.org/2021.11/) for quality control assessment and taxonomic classification, respectively.

### 2.9. AMOS-PCR and Bruce-Ladder PCR Assays

The multiplex AMOS-PCR includes species-specific primers, *B. abortus (*F-GAC GAA CGG AAT TTT TCC AAT CCC), *B. melitensis* (F-AAA TCG CGT CCT TGC TGG TCT GA), *B. ovis* (F-CGG GTT CTG GCA CCA TCG TCG GG), *B. suis* (F-GCG CGG TTT TCT GAA GGT GGT TCA), and reverse primer IS711 (R-TGC CGA TCA CTT AAG GGC CTT CAT) as described [22]. Four species-specific forward primers were used at a final concentration of 0.1 μM with 0.2 μM reverse primer IS711. PCR cycling conditions consisted of an initial denaturation at 95 °C for 5 min followed by 35 cycles of 95 °C for 1 min, 55.5 °C for 2 min, 72 °C for 2 min, and a final extension step at 72 °C for 10 min. Specific amplicon sizes were determined using agarose electrophoresis.

As described by [19,46], a multiplex Bruce-ladder PCR was performed to identify and distinguish between vaccine strains and field isolates of *Brucella* spp. The positive controls used were *B. abortus* bv 1 strain (REF 544, BCCN R4), *B. abortus* S19 (Design Biologix, Pretoria, South Africa), and *B. melitensis* Rev 1 (Onderstepoort Biological Products, Pretoria, South Africa). The amplified products were examined via electrophoresis in a 2% agarose gel and stained with ethidium bromide (0.03 μL/mL). The gel ran at 120 volts for 1 h. The gel was documented under UV light by a molecular imager (Bio-rad, ChemiDoc^TM^ XRS).

### 2.10. Statistical Analysis

Descriptive analysis was used to determine the frequency (percentage) of *Brucella* PCR positivity among the different variables (abattoir, throughput, animal species, and sex of the animal). The chi-squared or Fisher’s exact test was used to determine the association between PCR positivity, on the one hand, and each of the four variables, on the other hand, in univariate analyses. The four variables, regardless of their *p*-value, from univariate analyses were included in subsequent multivariable models. The multivariable analysis was conducted with generalized linear models with a stepwise backward elimination procedure and Akaike Information Criteria to determine the risk factors for *Brucella* infection. We determined the level of agreement between PCR and culture results for *Brucella* spp. using the Cohen’s kappa (*k*) test [47]. The kappa result was interpreted as follows: kappa ≤ 0 = no agreement; 0.01–0.20 = none to slight; 0.41–0.60 = moderate; 0.61–0.80 = substantial; and 0.81–1.00 = almost perfect agreement. Data analyses were performed using R statistical software version 4.21 [48], employing the packages “MASS” and “epiR”, and a 0.05 level of significance.

### 2.11. Ethical Considerations

Approvals from the Research and Animal Ethics Committees of the University of Pretoria (Ref: REC 028-22), section 20 of the Animal Diseases Act, (Act No. 35 of 1984) from the Department of Agriculture, Land Reform and Rural Development (DALRRD) were obtained. Appropriate health and safety precautions with risk assessments were followed throughout the collection and processing of the samples.

## 3. Results

### 3.1. Identification of Brucella spp. Directly from the Tissues Using 16S–23S Ribosomal DNA Interspacer (ITS) Region PCR Assay

Of the tissue samples from the 280 slaughtered cattle tested using the *Brucella* ITS-PCR, the frequency of detection was 33.57% (94/280) (Supplementary Figure S1). Of the 200 slaughtered sheep tested using ITS-PCR, the frequency of detection was 14.5% (29/200). Of the tissue samples from the 85 slaughtered pigs tested using the ITS-PCR, the frequency of detection was 4.71% (4/85).

### 3.2. Identification of Gram-Negative Isolates Using Gram Staining

Tissues from *Brucella* ITS-PCR positive animals (127/565) were included in culturing, after which round, smooth margin, translucent, and yellowish-white coloured colonies on modified CITA medium were examined using microscopy and staining. Of the 94 cattle tissues that tested positive with ITS-PCR, 41 *Brucella* isolates were identified based on Gram-negative cocco-bacilli using Gram staining and were positive with modified Ziehl–Neelsen staining. Fifteen (15) *Brucella* suspect cultures from 29 ITS-PCR-positive sheep tissues were identified using microscopy. Additionally, two *Brucella* cultures were observed from four ITS-PCR pig tissues based on microscopy. *Brucella* colonies were further subjected to several rounds of streaking and dilution to purify the colonies. Additional fast-growing Gram-negative bacteria were also observed on culture. *Spingomonas* was identified among other bacteria. The organism has an identical antibiogram, thus making it impossible to select and purify *Brucella* from this faster growing contaminant using antibiotics. None of the isolations could be purified and thus remained impure isolates, which we identified using AMOS-PCR.

### 3.3. Characterization of Brucella spp. Using AMOS-PCR Assay and Seropositivity

**Figure 2:**
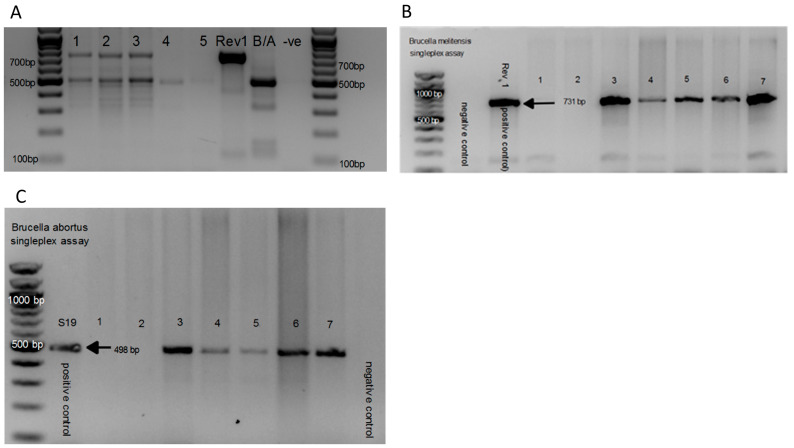
Amplification from *Brucella* isolates from sheep tissues using AMOS-PCR with 498 bp amplifying *B. abortus* target region and 731 bp amplifying the *B. melitensis* target region. (**A**) Multi-plex AMOS-PCR with a mixed infection of both *B. abortus* and *B. melitensis* isolated from the kidneys in lanes 1–3; *B. abortus* isolated from the liver in lane 4; negative control in lane 5 and 9 (-ve); *B. melitensis* Rev 1 and *B. abortus* positive controls in lane 6 and 7. (**B**) Single-plex *B. abortus*-specific primer of AMOS-PCR with negative water control and 731 bp *B. melitensis* PCR product using *B. melitensis* Rev 1 positive control; lanes 1–7 included tissues that were AMOS-negative in lanes 1 and 2; and lane 3–7 included mixed *B. abortus* and *B. melitensis* sheep isolates from 4 sheep (with the same animals repeated in lanes 3 and 7). (**C**) Single-plex *B. abortus*-specific primer of AMOS-PCR with negative water control and 498 bp *B. abortus* PCR product using *B. abortus* S19 positive control; lanes 1–7 included tissues that were AMOS-negative in lanes 1 and 2; and lane 3–7 included mixed *B. abortus* and *B. melitensis* sheep isolates from 4 sheep (with the same animals repeated in lanes 3 and 7).

Of the 41/94 (43.6%) *Brucella* suspect isolates observed on microscopy from ITS-PCR positive cattle tissues, AMOS-PCR characterized 38 as *B. abortus* and a mixed infection of both *B. abortus* and *B. melitensis* was observed in three cattle (Table 1). From the 15/29 (51.7%) *Brucella* suspect isolates from ITS-PCR positive sheep tissues, AMOS-PCR characterized 11 as *B. abortus* and a mixed infection of both *B. abortus* and *B. melitensis* was observed in four sheep (Figure 2A). Of the 2/4 (50%) *Brucella* suspect isolates from ITS-PCR positive pig tissues, AMOS-PCR characterized 1 as *B. abortus* and 1 as a mixed infection of *B. abortus* and *B. melitensis* (Table S1, supplementary data). The single-plex AMOS-PCR was used to separate and confirm the mixed infection of *B. abortus* and *B. melitensis* (Figure 2B,C). Using the AMOS-PCR and Bruce-ladder PCR assays, the isolates were distinguished from the 16S vaccine strain (Supplementary Figure S1).

Using AMOS-PCR, *Brucella* spp. was identified in 14.6% (41/280), 7.3% (15/200), and 2.4% (2/85) from cattle, sheep, and pig tissue collected from Eastern Cape abattoirs (Table 1). Seropositivity based on one or more serological tests (RBT, CFT, and/or iELISA) of *Brucella*-infected animals identified with AMOS-PCR consisted of 24.4% (10/41) cattle, 13.3% (2/15) sheep, and no pigs (Table 1). See Table 1 for the animals that were AMOS-PCR *Brucella* spp. infected and seronegative.

### 3.4. Brucella Isolation amongst Livestock Stratified by Tissue

*Brucella* isolation from ITS-PCR positive tissues and identified with AMOS-PCR stratified by cattle tissues was 33.0% (31/94) in the lymph nodes, 26.6% (25/94) in the liver, 21.3% (20/94) in the spleen, 20.2% (20/94) in the kidney, and 10.6% (10/92) in the tonsils. Regarding sheep tissues, *Brucella* isolates identified by AMOS-PCR were present in 37.9% (11/29) of the liver and kidney, 34.5% (10/29) of the spleen, and 27.6% (8/29) of the lymph nodes. No tonsil samples were collected from sheep as the abattoirs sell the head intact. *Brucella* isolates identified with AMOS-PCR from pigs were isolated from the tonsils (50%, 2/4) (Table 1).

### 3.5. Association between Brucella ITS-PCR Positivity and Predictor Variables

Three variables (abattoir, throughput, and animal species) out of four analysed in univariate analyses showed statistical significance (*p* ≤ 0.05) (Table 2). The four variables regardless of *p* value were included in a multivariable logistic regression model. After multivariable analysis, which followed a backward stepwise elimination procedure, only three variables (sex, species, and abattoir) out of the four comprised the final regression model (Table 3). The abattoir factor was a significant determinant for positivity amongst the specimens from different animal species. With abattoir B as the reference level, animals in abattoir D (39.1%; OR = 7.0, *p* = 0.00014), abattoir E (41.7%; OR = 5.13, *p* < 0.0001), and abattoir A (38.0; OR = 4.9, *p* < 0.0001) were more likely to be PCR positive for *Brucella* spp., while abattoir C (15.6%, OR = 0.91, *p* = 0.85) had a similar positivity rate (Table 2 and Table 3). There was an almost similar likelihood of Brucella positivity between male (21.1%) and female animals (23.9%) with an odds ratio between the two levels of 0.5 (Table 2 and Table 3).

### 3.6. Level of Agreement between PCR and Culture Results for the Detection of Brucella spp.

There was moderate agreement between the PCR and culture results (kappa = 0.57; 95% CI 0.47, 0.66; *p* < 0.0001). Of the 565 samples tested, 58 were positive with both PCR and culture methods, while 69 samples were positive on PCR but negative on culture. A total of 438 samples were negative with both methods.

### 3.7. Sequence Identification of Additional Gram-Negative and Gram-Positive Isolates from Culture

**Figure 3:**
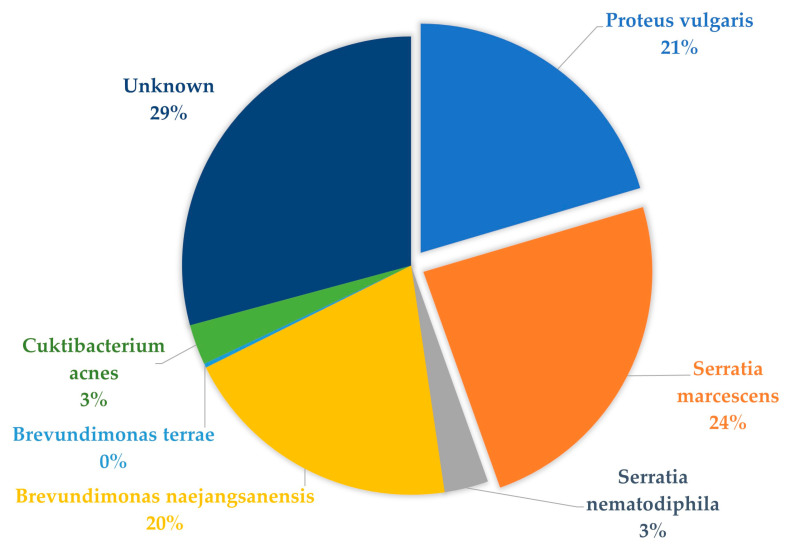
Sequencing identification of other bacterial organisms present in cultures from abattoir livestock tissues on modified CITA medium.

Faster-growing contaminants were a recurring hindrance to obtain pure *Brucella* isolates. To identify the contaminants, and, in doing so, attempting to improve the selective medium, isolates were submitted for sequencing. The following isolates were identified by nucleotide identity using QIIME2. *Proteus vulgaris* (21%), *Cuktibacterium acnes* (3%), *Brevundiminas terrae*, *Brevundimonas naejangsanensis* (20%), *Serratia nematodiphila* (3%), and *Serratia marcescens* (24%) were identified on culture from the livestock tissue samples (Figure 3).

## 4. Discussion

This study used samples available from abattoirs to investigate brucellosis, which allowed a polyphasic approach and thus serology, molecular, and bacteriology detection. Most studies only use serology, and few continue to obtain *Brucella* culture isolates. *Brucella*-specific PCR on tissues from livestock followed by culture and AMOS-PCR identification detected mainly *B. abortus* with a few mixed infections (*B. abortus* and *B. melitensis*) in 14.6% (41/280) of cattle, 7.5% (15/200) of sheep and 2.4% (2/85) of pig tissues collected from Eastern Cape Province abattoirs. This study demonstrated the value of the polyphasic approach, especially to identify the potential risk of brucellosis in asymptomatic animals with possible chronic infections.

This study isolated *Brucella* spp. from the liver, spleen, kidney, lymph nodes (mesenteric and mandibular), and tonsils of apparently healthy livestock from the abattoirs in the Eastern Cape province. Tissue samples from livestock including the liver, spleen, kidneys, lungs, and lymph nodes have previously been processed for the isolation of *Brucella* spp. [8,49]. In this study, of the 58 AMOS-PCR identified *Brucella* isolates, 19.0% (11/58) were seropositive using either RBT, CFT, or iELISA [43] with the majority being seronegative. The isolation of *Brucella* spp. from seronegative animals (see supplementary data) may be an indication of chronic infection in the animals [50], with these asymptomatic animals posing a risk of spreading the pathogen once they become pregnant or during breeding as *Brucella* will then start to replicate. Disease surveillance from live animals using serological tests is limiting and cannot detect latent or chronically infected animals and thus shows the value of the sample availability combined with molecular methods at abattoirs to determine the risk of contribution to disease spread and spillover [51].

Although the culture technique is not a sensitive procedure, bacterial isolation is considered as the gold standard for diagnosing *Brucella* spp. in humans and animals [52]. Aborted tissues from a *B. abortus* abortion episodic yield more than 10^14^ microbial organisms, which constitutes 10^5^ times the presumed infectious dosage of heifers vaccinated with S19 [53]. Hence, increased isolation of *Brucella* spp. on culture has been reported when sampling from aborted materials and vaginal swaps [54], as compared to tissues from asymptomatic and apparently healthy animals. Thus, *Brucella*-specific PCR was used for tissue screening before attempting low-sensitivity isolation, especially as the samples were collected from asymptomatic livestock. However, tissues were collected from organs with ER cells such as the spleen, liver, kidney, and lymph nodes as these are macrophage-rich tissues where *Brucella* organisms multiply and concentrate and thus increase culture sensitivity [8] and can only be collected from dead animals [23]. The *Brucella* spp. isolation frequency of cattle was higher in lymph nodes (31/94), followed by the liver (25/94) and spleen (20/94). The frequency of isolation of *Brucella* spp. in sheep was higher in the liver (11/29) and kidney (11/29), followed by the spleen (10/29). *Brucella* isolates were only recovered from the tonsils in 2/4 pigs. *Brucella* spp. was isolated and detected with AMOS-PCR from the lymph nodes, liver, spleen, and kidney samples from animals showing no clinical signs of brucellosis infection. Thus, this suggests that the above-mentioned tissues may be utilized for brucellosis screening purposes and diagnostics in slaughtered abattoir animals. This study also highlights improved assessment standards and procedures that may result from routine sampling, such as obtaining tonsils from monogastric animals and the liver, kidneys, and spleen from ruminants.

This study further shows the presence of fast-growing contaminants which makes the isolation of low-concentration *Brucella* from asymptomatic animals impossible despite various attempts. It has been reported that *Brucella* isolation from vaginal secretions, placenta, foetal tissues, milk, and semen from animals are normally impaired by contaminants that overgrow the slow-growing brucellae, even on selected media [8]. The presence of these other fast-growing Gram-negative bacteria on culture affects the growth of *Brucella* spp. through competitive inhibition, thus resulting in impure/contaminated isolates. The present study reports the isolation of other pathogenic organisms such as *Proteus Vulgaris*, *Serratia marcescens*, and *Brevundimonas naejangsanensis. Proteus vulgaris* has been reported as a zoonotic infection, which is mainly known for causing wound and urinary infections in humans [55]. Previous researchers have reported *S. marcescens* as a common cause of mastitis and early abortions in cows [56,57]. *Brevundimonas naejangsanensis* is an environmental Gram-negative bacterium, which has been isolated from the soil [58]. The risk of zoonotic diseases is increased by the isolation of potentially harmful foodborne pathogens such as *Brucella* spp. and *P. vulgaris* [59], from apparently healthy abattoir livestock. Microbial contamination of the abattoir meat may occur during the exsanguination process, particularly if a sterile environment is not maintained [60]. Based on our study, it was observed that the butcher only washes their knife to remove the excessive amount of blood and not to avoid contamination from one animal to the other. It was also observed that the operators clean/spray the floors frequently for blood removal. However, this process allows contaminated water/blood to splash onto the meat. In [60], the authors reported the blood removal procedure on the floor as being unhygienic. To reduce these contaminants, it is advisable to surface-sterilize the tissues before culturing, which can reduce these contaminants.

In this study, on gross pathological examination, yellowish-white lesions, discoloration/bruises, abscesses, and cysts were observed on some cattle tissues. This included the mesenteric lymph nodes, skin, liver, and the spleen. The presence of lesions in the mesenteric lymph nodes can indicate *Mycobacterium tuberculosis* complex infection. As reported by a similar study conducted in the Eastern Cape abattoirs, the presence of nodular lesions was observed in 162 cattle lymph node samples with visible inflammation [61]. Their study reported the isolation of *Mycobacterium bovis* and *M. tuberculosis* [61]. Feedlot cattle may develop liver abscesses as a result of vigorous grain-feeding programs, which are also influenced by a number of nutritional and management factors [62]. Our findings are in agreement with other studies which identified major causes of offal and carcass condemnation in the Eastern Cape abattoirs including tongue and spleen abscesses, bruises, actinobacillosis, heart and kidney cysts, inflammatory conditions, and improper evisceration [63]. However, the underlying causes of the conditions remain unknown. Due to the tissue condemnation and decreased meat yield, the presence of pathological evidence on the tissue has a major economic impact on the animal industry [62] and increases the risks of zoonotic infections to humans.

Multivariable analyses showed that sheep (14.5%; OR = 5.6, *p* = 0.0043) and cattle (33.6%; OR = 17.1, *p* < 0.0001) were significantly more likely to be AMOS-PCR-positive for *Brucella* species compared to pigs (4.7%). The current study reports the isolation of *Brucella* spp. in 43.6% (41/94) cattle, 51.7% (15/29) sheep, and 50% (2/4) pig samples using AMOS-PCR, which only detects *B. abortus* bv 1, 2, and 4, *B. melitensis* bv 1–3, *B. ovis*, and *B. suis* bv 1. A similar study conducted in the Eastern Cape Province reported an increased isolation of *Brucella* spp. from cattle (62.3%) as compared to goats (25.4%) and sheep (12.3%), also using AMOS-PCR. The current bovine brucellosis scheme includes the mandatory vaccination of heifers aged 4–8 months using 16S vaccine, serological testing, and surveillance of high-risk farms, particularly dairy and breeding cattle with suspected or proven brucellosis infections [64,65]. However, the participation of the farmers is voluntary and self-funded, thus negatively affecting the role and importance of early vaccination. None of the *B. abortus* isolates from the livestock tissue were the S19 vaccine strain. This study indicates an almost similar likelihood of *Brucella* positivity between male (21.1%) and female animals (23.9%), with an odds ratio between the two levels of 0.5. The abattoirs (except abattoir C), species, and sex were significant determinants for positivity in our study with a *p* ≤ 0.05. The *Brucella* positivity in male animals may be due to high exposure of the bacteria or through the consumption of milk from infected females. An increased positivity was observed from low-throughout abattoirs (39.1%) as compared to high-throughput abattoirs (21.8%). As reported by [41], an increased sero-positivity and isolation of *Brucella* spp. was also observed from low-throughput abattoirs as compared to high-throughput abattoirs in Gauteng Province. This may be because low-throughput abattoirs receive animals from the local community alongside animals from the same herd or animals grazing together, thus increasing the possibility of transmission between each other.

Brucellosis is a controlled zoonotic infection in animals and a notifiable disease in humans in SA [66]. The infection is a major public health challenge and still predominant as a neglected endemic zoonosis requiring proactive considerations in numerous communities worldwide [67]. Serological tests have been used to detect brucellosis throughout SA in bovine. However, brucellosis outbreaks have been reported mainly in the central and highveld regions [68]. The brucellosis scheme in SA is focused on bovine, and, from this study, *B. abortus* was the dominant species detected with AMOS-PCR in *Brucella*-infected animals. *Brucella abortus* was not only detected in bovine but also sheep and pigs, which indicates spill over to these species in SA. A previous study conducted in the Eastern Cape, reported the isolation of *B. abortus* in cattle, sheep, and goats, whereas the isolation of *B. melitensis* was observed in sheep and goats [69]. As reported by [41], the first case in SA of *B. melitensis* in cattle was isolated from abattoirs in Gauteng Province. The current study reports the isolation of *B. melitensis* from abattoirs cattle in the Eastern Cape Province. Serological tests cannot differentiate between *Brucella* species, therefore, brucellosis seropositive bovines are presumed to be *B. abortus*, while seropositive sheep and goats are presumed to be infected with *B. melitensis*. Mixed *B. abortus* and *B. melitensis* infections were also detected in all livestock in this study and need further investigations. Despite the tremendous efforts of the SA government in the eradication of infection, an increased number of reports continue to indicate the presence of brucellosis in livestock in SA [41,69]. Surveillance schemes in countries where brucellosis has been eradicated mainly focus on the vaccination of livestock as well as the test and slaughtering schemes of all relevant species [70], unlike SA, which focuses only on high-risk bovines [71]. Brucellosis eradications takes decades, and it is a costly exercise [72]. In endemic countries such as SA, serological tests will have their limitations due to chronically infected animals since the antibody level is below detection in these animals. However, serological tests will identify some infected animals, but the results in this study indicate that testing should be expanded to all bovine and to other livestock species as well, especially sheep and goats, to increase the detection of brucellosis.

## 5. Conclusions

This study has demonstrated the importance of multiple tests in the diagnosis and surveillance of brucellosis, as is evidenced by the isolation and identification of *B. abortus* and *B. melitensis* from seropositive but mainly seronegative asymptomatic livestock. The use of only serological tests in chronic infected animals results in false negative results. This study demonstrates the value of the polyphasic approach using the molecular method in combination with samples from abattoirs, especially to identify the potential risk of brucellosis in asymptomatic animals with possible chronic infections. This study also emphasizes refined evaluation criteria, and processes could come from routine sampling, i.e., collecting the liver, kidneys, and spleen from ruminants and tonsils from slaughtered animals. Abattoirs prove to be a valuable surveillance resource as their tissues are easily accessible post-slaughter. More data included from such sites would allow for a much clearer epidemiological picture of brucellosis in provinces across SA. This could, in turn, provide better data with which to plan targeted surveillance for both *B. abortus* and *B. melitensis* infections in livestock and to make effective management decisions against this devastating herd disease.

## 6. Limitations of the Study

The isolation of *Brucella* spp. was recovered from the livestock samples. However, due to the increased growth of other fast-growing Gram-negative bacteria, impure cultures were observed. The Bruce-ladder PCR assay requires highly concentrated *Brucella* DNA to amplify the multiple targets of this assay. Mixed infections of *B. abortus* and *B. melitensis* were observed in all the species (cattle, sheep, and pigs). However, due to the confluent growth of contaminants, the mixed *Brucella* spp. could not be isolated separately. Further investigation, which will be possible in a larger study, is recommended. This could include the surface sterilization of tissues to reduce the growth of other organisms, thus allowing the *Brucella* spp. to grow confluently.

## Figures and Tables

**Table 1:** Brucellosis characterization of slaughtered livestock using 16–26S ribosomal interspacer region (ITS)-PCR, *Brucella* isolation identified with AMOS-PCR stratified by tissue and serological information using the Rose Bengal test (RBT), complement fixation test (CFT), and iELISA (latter from [43]).

Species	ITS-PCR-Positive Animals (%)	Culture AMOS-PCR Animals (%)	Culture-Positive Animals Identified with AMOS-PCR from ITS-PCR-Positive Tissue (%)	Number Positive Tissues per Animal Species					Seronegative (RBT, CFT and iELISA) and Culture-Positive Animals	*Brucella* Culture and Seropositive Animals			
				Liver	Spleen	Kidney	Lymph Nodes	Tonsils		RBT	ELISA	RBT and iELISA	RBT, iELISA, and CFT
Cattle	94/280 (33.6%)	41/280 (14.6%)	41/94 (43.6%)	25/94 (26.6%)	20/94 (21.3%)	19/94 (20.2%)	31/94 (33.0%)	10/94 (10.6%)	31/41 (76.6%)	7/41 (17.1%)	4/41 (9.8%)	2/41 (4.9%)	1/41 (2.4%)
Sheep	29/200 (14.5%)	15/200 (7.5%)	15/29 (51.7%)	11/29 (37.9%)	10/29 (34.5%)	11/29 (37.9%)	8/29 (25.6%)	-	13/15 (86.7%)	2/15 (13.3%)	0/15	0/15	0/15
Pigs	4/85 (4.7%)	2/85 (2.4%)	2/4 (50.0%)	0/4	0/4	0/4	0/4	2/4 50.0%)	2/2 (100%)	0/15	0/15	0/15	0/15

**Table 2:** Descriptive and univariate analyses to determine the association between various factors and occurrence of *Brucella* spp. in the tissue was determined using ITS-PCR.

Variable	Level	Number of Animals Positive for *Brucella* spp. (%)	*p*-Value
Abattoir			<0.0001
	Abattoir A (n = 50)	19 (38.0)	
	Abattoir B (n = 344)	48 (14.2)	
	Abattoir C (n = 45)	7 (15.6)	
	Abattoir D (n = 23)	9 (39.1)	
	Abattoir E (n = 103)	43 (41.7)	
Throughput			0.05078
	High (n = 542)	118 (21.8)	
	Low (n = 23)	9 (39.1)	
Animal species			<0.0001
	Cattle (n = 280)	94 (33.6)	
	Pig (n = 85)	4 (4.7)	
	Sheep (n = 200)	29 (14.5)	
Sex	Female (n = 276)	66 (23.9)	0.4245
	Male (n = 289)	61 (21.1)	

**Table 3:** Multivariable analysis.

Variable	Category	Odds Ratio (CI)	*p*-Value
Abattoir	Abattoir B (ref)		
	Abattoir A	4.89 (2.26, 10.57)	<0.0001
	Abattoir C	0.91 (0.36, 2.30)	0.8495
	Abattoir D	7.02 (2.57, 19.15)	0.000142
	Abattoir E	5.13 (2.92, 8.99)	<0.0001
Species			
	Pig (ref)		
	Cattle	17.09 (5.66, 51.61)	<0.0001
	Sheep	5.59 (1.71, 18.29)	0.0043
Sex			
	Male (ref)		
	Female	0.54 (0.33, 0.89)	0.016

## Data Availability

All the relevant data and supplementary information are contained within the paper.

## Supplementary Materials

The following supporting information can be downloaded at https://www.mdpi.com/article/10.3390/microorganisms12010223/s1. All supplementary materials used in this study are attached as a separate file; Table S1: Molecular and serological identification of *Brucella* spp. in livestock.; Figure S1: Gel electrophoresis of Bruce-Ladder PCR amplification to differentiate the field strains from vaccine strains. Lanes 1–5 and 10 show amplification of *B. abortus*, lanes 6–8 show amplification of *B. melitensis*, lane 9 show Rev 1 positive and lane 11 and 12 show negative controls.
