# Supplementary material for: Polyphasic Characterization of Brucella spp. in Livestock Slaughtered from Abattoirs in Eastern Cape, South Africa
Source: Microorganisms. 2024 Jan 22;12(1):223. doi: 10.3390/microorganisms12010223 (PMC10819803; doi:10.3390/microorganisms12010223)
Supplement: Supplementary file 1 [file microorganisms-12-00223-s001.zip › microorganisms-2752187-supplementary.pdf]

Supplementary Materials

Polyphasic Characterization of *Brucella* spp. in Livestock Slaughtered from Abattoirs in Eastern Cape, South Africa

Koketso Desiree Mazwi [1,*], Francis Babaman Kolo [1], Ishmael Festus Jaja [2,3], Charles Byaruhanga [1,4], Ayesha Hassim [1] and Henriette van Heerden [1,*]

[1] Department of Veterinary Tropical Diseases, Faculty of Veterinary Science, University of Pretoria,
Onderstepoort 0002, South Africa; kolofrancis@hotmail.com (F.B.K.); cbyaruhanga27@yahoo.com (C.B.); ayesha.hassim@gmail.com (A.H.)

[2] Department of Livestock and Pasture Science, Faculty of Science and Agriculture, University of Fort Hare, Alice 5700, South Africa; ijaja@ufh.ac.za

[3] Department of Agriculture and Animal Health, University of South Africa, Roodepoort, Johannesburg 1709, South Africa

[4] National Agricultural Research Organisation, Entebbe P.O. Box 259, Uganda

* Correspondence: desireemazwi@gmail.com (K.D.M.); henriette.vanheerden@up.ac.za (H.v.H.)

Table S1: Molecular and serological identification of *Brucella spp.* in livestock.

Batch number (per collection)	Abattoirs	Positive direct Tissue screening (ITS PCR)	Culture isolates (AMOS PCR)
	Abattoir A pigs	0/20	-
	Abattoir B cattle	26/30	5 - *B. abortus*
Batch 1	Abattoir B sheep	4/20	3 – Mixed infections (*B. abortus* and *B. melitensis*)
	Abattoir C cattle	7/30	7 – *B. abortus*
	Abattoir D sheep	9/20	1 – *B. abortus*
	Abattoir E cattle	13/40	3 – *B. abortus*<br>1 – Mixed infections (*B. abortus* and *B. melitensis*)
Batch 2	Abattoir A cattle	19/30	6 – *B. abortus*
	Abattoir B cattle	1/20	1 – Mixed infections (*B. abortus* and *B. melitensis*)
	Abattoir C pig	0/15	-
	Abattoir D cattle	0/3	-
	Abattoir E sheep	15/20	10 – *B. abortus*<br>1 – Mixed infections (*B. abortus* and *B. melitensis*)
	Abattoir E cattle	11/27	10 – *B. abortus*<br>1 – Mixed infections (*B. abortus* and *B. melitensis*)
Batch 3	Abattoir B cattle	17/100	7- *B. abortus*
	Abattoir B sheep	1/140	0
	Abattoir B pig	0/34	-
	Abattoir E pig	4/16	1 – *B. abortus*<br>1 – Mixed infections (*B. abortus* and *B. melitensis*)

*The numbers in this table represent carcasses

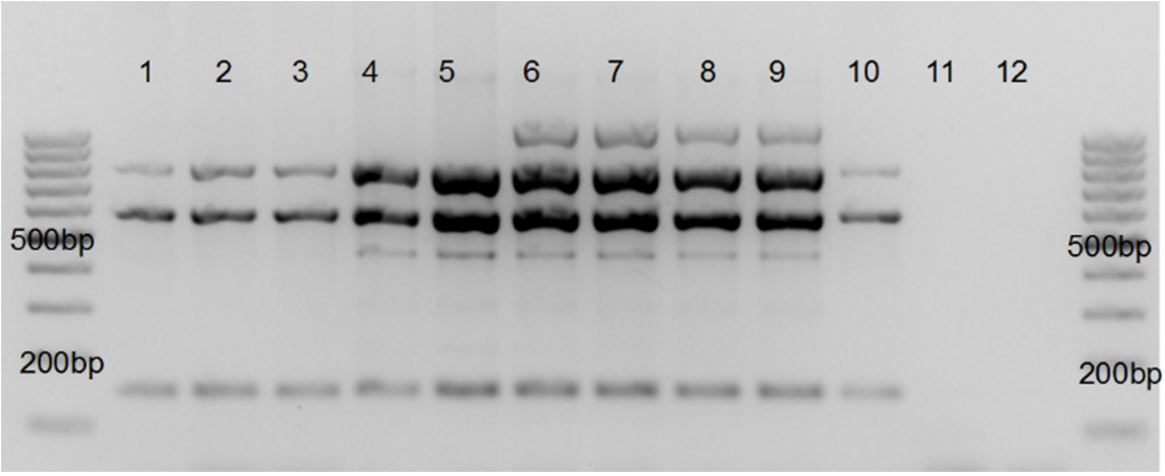

Figure S1: Gel electrophoresis of Bruce-Ladder PCR amplification to differentiate the field strains. Lanes 1-5 and 10 show amplification of *B. abortus,* lanes 6-8 show amplification OF *B. melitensis,* lane 9 show Rev 1 positive and lane 11 and 12 show negative controls.
